# Enhanced classification and severity prediction of major depressive disorder using acoustic features and machine learning

**DOI:** 10.3389/fpsyt.2024.1422020

**Published:** 2024-09-17

**Authors:** Lijuan Liang, Yang Wang, Hui Ma, Ran Zhang, Rongxun Liu, Rongxin Zhu, Zhiguo Zheng, Xizhe Zhang, Fei Wang

**Affiliations:** ^1^ Laboratory of Psychology, The First Affiliated Hospital of Hainan Medical University, Haikou, Hainan, China; ^2^ Early Intervention Unit, Department of Psychiatry, Affiliated Nanjing Brain Hospital, Nanjing Medical University, Nanjing, China; ^3^ Hainan Provincial Institute of Mental Health, Hainan Provincial Anning Hospital, Haikou, Hainan, China; ^4^ School of Information and Communication Engineering, Hainan University, Hainan, China; ^5^ School of Information Engineering, Hainan Vocational University of Science and Technology, Hainan, China; ^6^ School of Biomedical Engineering and Informatics, Nanjing Medical University, Nanjing, China

**Keywords:** major depressive disorders, vocal acoustic features, classification, prediction, model

## Abstract

**Background:**

Previous studies have classified major depression and healthy control groups based on vocal acoustic features, but the classification accuracy needs to be improved. Therefore, this study utilized deep learning methods to construct classification and prediction models for major depression and healthy control groups.

**Methods:**

120 participants aged 16–25 participated in this study, included 64 MDD group and 56 HC group. We used the Covarep open-source algorithm to extract a total of 1200 high-level statistical functions for each sample. In addition, we used Python for correlation analysis, and neural network to establish the model to distinguish whether participants experienced depression, predict the total depression score, and evaluate the effectiveness of the classification and prediction model.

**Results:**

The classification modelling of the major depression and the healthy control groups by relevant and significant vocal acoustic features was 0.90, and the Receiver Operating Characteristic (ROC) curves analysis results showed that the classification accuracy was 84.16%, the sensitivity was 95.38%, and the specificity was 70.9%. The depression prediction model of speech characteristics showed that the predicted score was closely related to the total score of 17 items of the Hamilton Depression Scale(HAMD-17) (r=0.687, P<0.01); and the Mean Absolute Error(MAE) between the model’s predicted score and total HAMD-17 score was 4.51.

**Limitation:**

This study’s results may have been influenced by anxiety comorbidities.

**Conclusion:**

The vocal acoustic features can not only effectively classify the major depression and the healthy control groups, but also accurately predict the severity of depressive symptoms.

## 1 Background

World Health Organization (WHO) data showed that the global prevalence of depression was approximately 15%-18%. By 2030, depression may become the leading cause of disease burden Patel et al. (1). The epidemiological survey revealed that the prevalence of depression was 3.4% in China Huang et al. (2). Major depressive disorder was characterized by high rates of recurrence, suicide risk, and social burden. Limited healthcare resources and inadequate recognition of major depressive disorder present some challenges, such as delayed identification and diagnosis and unsatisfactory treatment outcomes. The lack of timely assessment and diagnosis of major depressive disorder leads to delayed intervention and treatment, higher relapse rates, poor treatment efficacy, and increased treatment costs, more burden on individuals, families, and society.

Emotion is a complex psychological phenomenon accompanied by subjective experiences and distinctive vocal and acoustic behavior. Previous research has shown the correlation between psychiatric disorders and abnormal acoustic features in speech. For instance, bipolar disorder and schizophrenia feature atypical vocal acoustic characteristics Weiner et al. (3)Compton et al. (4)Faurholt-Jepsen et al. (5). The primary symptoms of major depressive disorder are persistent feelings of sadness, loss of interest, and psychomotor retardation. It also features related abnormal vocal and acoustic features, such as slower speech rate, reduced loudness, decreased intonation variation, and prolonged pauses Zhang et al. (6)Yang et al. (7). Furthermore, as the severity of depressive symptoms increases, objective vocal acoustic features, such as Average Weighted Variance (AWV), show a significant decrease, which indicates a narrower range of vocal variability and smoother acoustic trajectories Cummins et al. (8)Drugman et al. (9).

Several studies support the significant effectiveness of Mel Frequency Cepstral Coefficients (MFCC) in predicting and distinguishing depression. The density of the MFCC feature space is positively associated with the severity of depressive symptoms, which indicates that as the symptoms worsen, the MFCC feature space becomes more densely clustered Cummins et al. (8). Since MFCC has different types, researchers have conducted various speech reading or expression tasks for both the depression group and healthy group. Further analysis using Receiver Operatorating Characteristic (ROC) curves showed a sensitivity of 77.8%, specificity of 86.1%, and accuracy of 81.9% for MFCC2, indicating that MFCC is an effective vocal acoustic feature for identifying depression Taguchi et al. (10).

However, the relationship between depressive symptoms and vocal acoustic features still requires further investigation. Most previous studies assumed a linear correlation between acoustic features and depressive symptoms, primarily analyzing prosody features Mundt et al. (11). In contrast, research on the spectral features related to depression has been relatively scarce. Research on college students with depression has indicated that the model, including both prosody and spectral features, could have better potential classification and predictive power Wang et al. (12). The previous research demonstrated that vocal acoustic features could be important potential biomark of depression Cornet and Holden (13)Sverdlov et al. (14).Therefore, the purpose of this research is to further explore the relationship between vocal acoustic features and MDD, build classification and prediction Model through deep learning.

## 2 Methods

### 2.1 Ethics statement

Ethical approval for this study was obtained from the Nanjing Medical University Ethics Committee (Approval No. 2020-KY027-01). All participants were required to sign a written informed consent form, either personally or through their legal guardians, prior to beginning the study.

### 2.2 Participants

The present study included 120 participants aged 16-25(Due to incomplete recording of speech, 2 participants of MDD group have been excluded in this research). 64 participants diagnosed with Major Depressive Disorder (MDD) were assigned to the MDD group, and 56 others were placed in the Healthy Control (HC) group. The participants were matched for gender and age, and all were of Han ethnicity. All participants had at least a junior school education level and could read Chinese materials. They had no history of disorders affecting vocal acoustic features or nasal, pharyngeal, or laryngeal conditions that could influence vocal acoustic sound production. The HC group had no history of Axis I diagnoses according to Diagnostic and Statistical Manual of mental disorder – fourth edition (DSM-IV) criteria and no first-degree relatives with such histories. They had not undergone any psychiatric treatments during the month prior to the study. The MDD group was identified through structured interviews, confirming their meeting the criteria for a depressive episode diagnosis according to DSM-IV and excluding other Axis I diagnoses and severe suicidal risk.

### 2.3 Depression measurement

The diagnosis of depressive episodes in the MDD group was evaluated based on the diagnostic criteria for depression outlined in the Diagnostic and Statistical Manual of mental disorder – fourth edition (DSM-IV). The Structured Clinical Interview for DSM Disorders (SCID), the psychiatric structured clinical interview, was utilized to assess whether the MDD group met the criteria for a depressive episode diagnosis. Additionally, the non-patient version of the SCID was used to evaluate the clinical condition of the HC group participants.

The Hamilton Depression Scale-17 (HAMD-17) have been widely utilized by clinicians and researchers to evaluate the severity of depressive symptoms. It consists of a total of 17 items, with each item being rated on a 5-point scale ranging from 0 to 4. The interpretation of the total score is as follows: Total score ≤ 7: Normal;7 < Total score ≤ 17:Possible mild depression;17 < Total score ≤24: Possible moderate depression; Total score > 24: Possible MDD. Considering the comorbidity of depression and anxiety, this study also used HAMA-14 to evaluate anxious symptoms. It consists of 14 items, with each item being also rated on a 5-point scale ranging from 0 to 4. Total score ≤ 7: normal; 7 < total score ≤ 14 points: Possible anxiety; 14 < total score ≤ 21: anxiety; 21 < total score ≤ 29: obvious anxiety; Total score > 29 points: severe anxiety.

### 2.4 Collection of speech data

During the experiment, participants were instructed to sit comfortably on the chair. The recording device was placed approximately 5-10cm away from the participants, and they were asked to maintain a stable body posture throughout the experiment. The experimental instruction was as follows: “We will now invite you to read the following passage, ‘Life Like a Summer Flower’ (see Appendix), which will take approximately 2 minutes. Please read it freely, using your usual speaking style and pace, without any specific emotional expression.” To further standardize the experimental material, 45 participants were recruited before the formal experiment to assess the emotional valence of the passage with the 5-point Likert scale. The scale ranged from 1 (very negative) to 5 (very positive). The results indicated an emotional valence of 2.9 for the passage, indicating a neutral emotional tone. All experiments were conducted in separate rooms located in wards or laboratories with good soundproofing. Non-authorized personnel were instructed to avoid entering the experimental area during the experiment to prevent any interference.

### 2.5 Features extraction

The raw data of audio data primarily involves two steps: endpoint detection and format normalization. Once the audio data was preprocessed, this study conducted feature extraction on all the audio recordings of the passages. All audio materials were uniformly converted to wav format, mono, and then transcoded audio sampling to 16000HZ.

For feature extraction, this study utilized the open-source speech processing algorithm Covarep to extract 74-dimensional features, while an additional 46 features were extracted using Python programming functions (**Supplementary Table 1**). Furthermore, the open-source algorithms in Covarep were employed to calculate 10 statistical measures such as maximum, minimum, median, mean, standard deviation, kurtosis, skewness, regression slope, regression intercept, and regression coefficient R2 of these 120 vocal acoustic features. These statistical dimensions were utilized to describe the characteristics of the vocal acoustic features. Consequently, 1200 high-level statistical functions were computed for each sample Wang et al. (12).

### 2.6 Data analysis

Independent-sample t- and chi-square tests χ^2^ were utilized to analyze the difference in demographic data and clinical information between groups using SPSS Statistics Version 23 (IBM, Armonk, NY).

This research used Python (2021.1.1x64) and Pearson correlation analysis for feature selection. The vocal acoustic features of the samples were correlated with the severity of depressive symptoms, a heatmap of the correlation coefficients was plotted, and the vocal acoustic features with significant correlations (p < 0.01) were excluded. Based on feature dimensionality reduction, univariate correlation analysis was carried out between key speech feature sets, such as Mel-cepstral Efficient Phase (MCEP), MFCC, MFCC_deltas, MFCC_delta_deltas, Formant, and the severity of depressive symptoms.

Pearson correlation analysis was utilized to explore the correlation between the 1200 vocal acoustic features and the severity of depressive symptoms for all participants. A heatmap was generated to visualize the correlation coefficients of these features. The features with a significance level of p < 0.01 were selected as important.

In this study, Neural Architecture Search was conducted to explore the optimal four-layer neural network model for predicting the HAMD-17 scores. A binary classification algorithm was utilized to analyze the effectiveness of the critical vocal acoustic speech features in discriminating the MDD from HC. The neural network’s structure was obtained using a grid search in the parameter space. The input variables of the neural network were significantly correlated with vocal acoustic features, and the output variables of the classification model were 1 (representing MDD) and 0 (representing HC). The validity of the classification model was evaluated by the average area under the curve (AUC) of the ROC curve, and the area of vocal acoustic features under the curve was estimated. The five-fold cross-validation approach was utilized to reduce the over-fitting of training data, and scatter plots indicated the correlation between predicted scores and actual scores. Additionally, the mean absolute error was used to evaluate the accuracy of the predictive model.

## 3 Results

### 3.1 Statistical results of demographic and clinical data

All participants were of Han ethnicity. Independent samples t-tests revealed no significant differences in age and gender between the MDD and HC groups (p < 0.05). However, significant statistical differences were found in education level and scores on the depression and anxiety clinical scales between the MDD and HC groups. **Table 1** presents all participants’ detailed demographic and clinical information.

**Table 1:** Demographic data and clinical information of MDD and HC.

	MDD(N=64)	HC(N=56)	*t/*χ^2^	P
Gender (male/female)	14/50	11/45	0.09	0.76
Age	18.83(2.95)	19.26(1.22)	1.02	0.30
Education year	11.75(2.42)	13.60(1.41)	4.73	<0.001
HAMD-17 Total Scores	22.27(6.41)	2.69(2.01)	21.94	<0.001
Somatic anxiety (Factor)	6.71(2.70)	0.62(0.70)	16.37	<0.001
Psychic anxiety (Factor)	6.48(2.09)	0.87(0.93)	18.54	<0.001
Core depressive (Factor)	9.31(2.35)	1.19(1.03)	23.91	<0.001
Anorexia (Factor)	1.39(1.21)	0.07(0.25)	7.99	<0.001
HAMA-14 Total Scores	23.15(6.69)	1.55(1.63)	23.54	<0.001

Continuous variables are expressed as mean (standard deviation) and categorical variables as number/number.

### 3.2 Dimensionality reduction of vocal acoustic features

**Figure 1:**
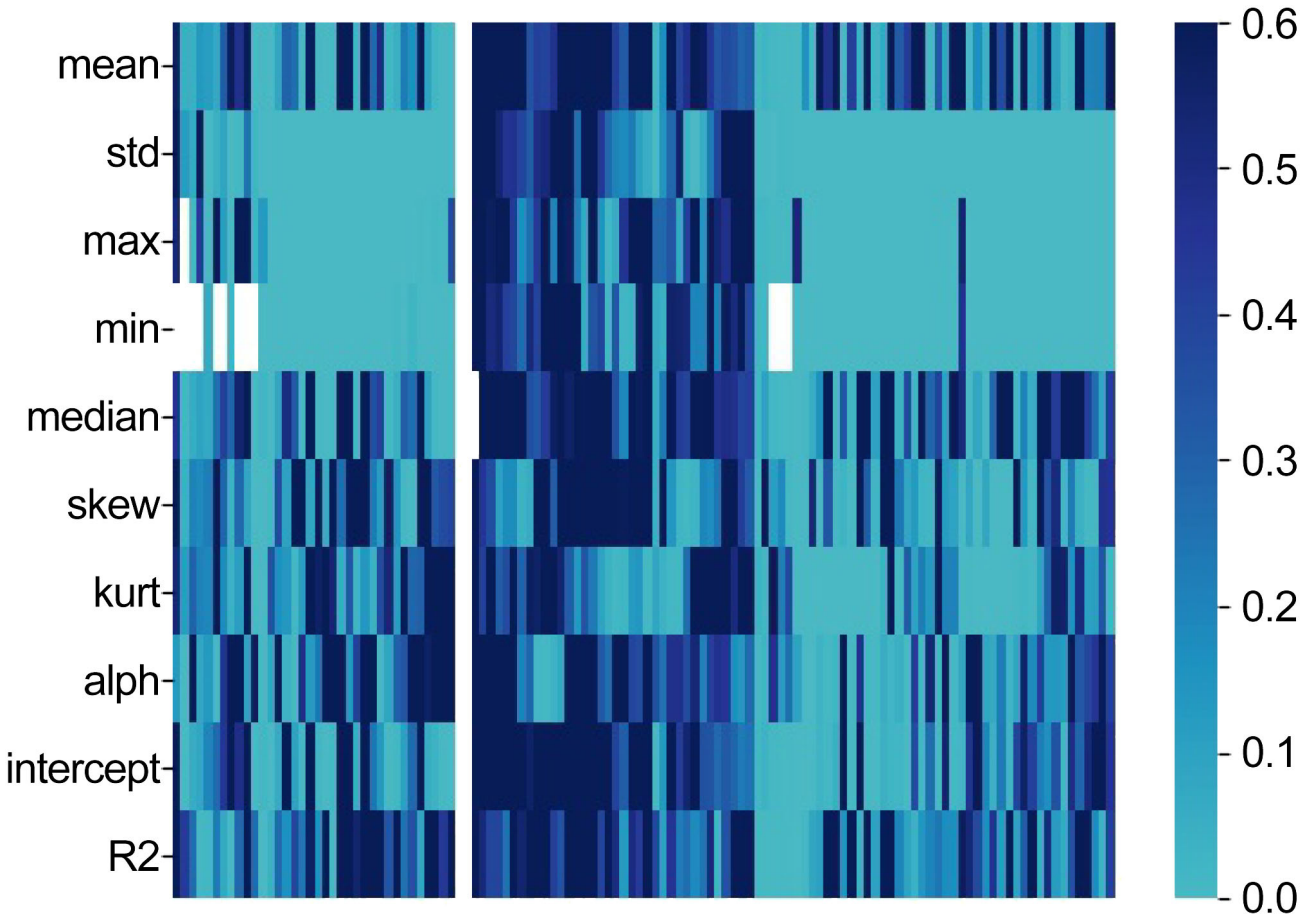
Heatmap (The horizontal axis represents the HAMD-17 score).

In the analysis, a Pearson correlation was conducted between the 1200-dimensional acoustic speech features, derived from the 10 statistical measures including maximum value, minimum value, median, mean, variance, kurtosis, skewness, regression slope, regression intercept, and regression coefficient, and the severity of depressive symptoms measured by the HAMD-17 total score. The results indicated that 331 acoustic speech features exhibited a significant correlation (p < 0.01) with the severity of depressive symptoms in all participants(as shown in **Figure 1**).

### 3.3 Relationship between abnormal acoustic features and severity of symptoms in major depressive disorders

#### 3.3.1 Relationship between abnormal related Mel Cepstral Coefficients features and depressive symptoms

**Figure 2:**
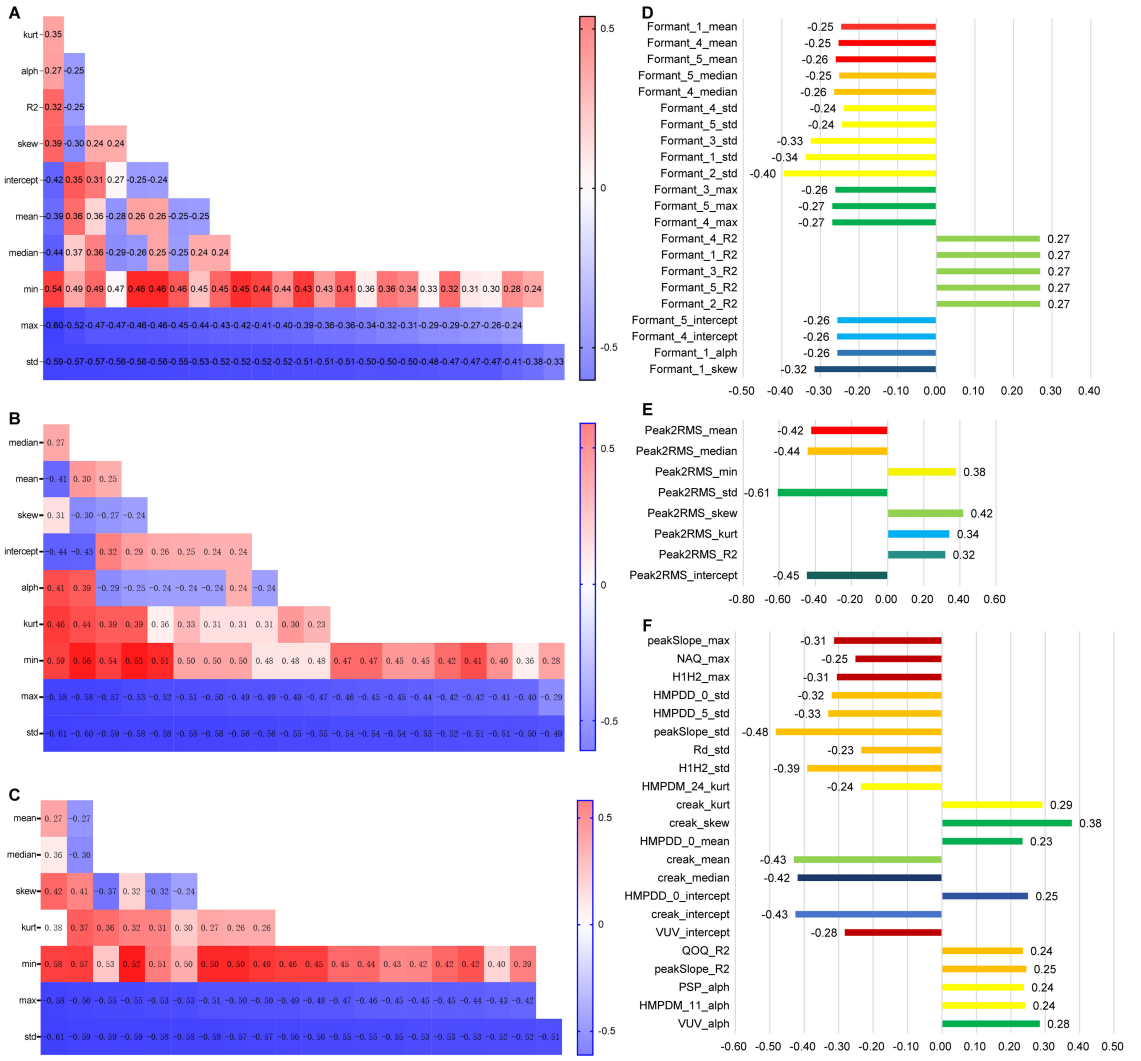
Analysis of the Correlation between Speech Features and Depression (**A–C** horizontal axes represent the HAMD-17 score, while **D–F** horizontal axes represent the correlation coefficient between speech and HAMD-17 score).

The results of the Pearson correlation analysis revealed that 104 dimensions of the Mel Cepstral Coefficients (MCEP) were significantly correlated with the HAMD-17 total score. When compared to the HC group, the MDD group exhibited significantly lower variability in the MCEP (as shown **Figure 2A**). Additionally, 96 acoustic speech features related to Mel Frequency Cepstral Coefficients deltas (MFCC_deltas) showed significant correlations with the HAMD-17 total score. Compared to the HC group, the MDD group demonstrated significantly reduced variability in the abnormal dynamic spectral features of MFCC_deltas (as show **Figure 2B**). Furthermore, 77 acoustic speech features related to MFCC_delta_deltas exhibited significant correlations with the HAMD-17 total score. Compared to the HC group, the MDD group displayed significantly reduced spectral dynamic changes in the abnormal speech acoustic features of MFCC_delta_deltas (as shown **Figure 2C**).

#### 3.3.2 Relationship between abnormal related formant features and depressive symptoms

In total, 24 acoustic speech features related to Formants were found to be significantly correlated with the HAMD-17 total score. Compared to the HC group, the MDD group exhibited significant abnormalities in the resonance peaks of speech acoustic features. Specifically, the MDD group demonstrated significantly decreased and less variable resonance peaks in the formants (as shown in **Figure 2D**).

#### 3.3.3 Relationship between abnormal related Peak2RMS features and depressive symptoms

In total, 8 dimensions of the Peak-to-RMS (Peak2RMS) acoustic speech features were significantly correlated with the HAMD-17 total score. Compared to the HC group, the MDD group exhibited significant decreases in the median and standard deviation and significant increases in the minimum value, kurtosis, and skewness. This result indicates that the abnormal speech acoustic features in MDD are characterized by significant decreases in overall loudness and volume variability (as shown **Figure 2E**).

#### 3.3.4 Relationship between other abnormal vocal acoustic features and depressive symptoms

In total, 22 acoustic speech features, including Voiced/Unvoiced (VUV), Quotient of the First Two Formants (QOQ), Spectral Tilt (Rd), Peak Slope (peakSlope), Number of Aspirated Quanta (NAQ), Harmonic Model Phase Deviation Mean (HMPDM), Harmonic Model Phase Deviation (HMPDD), Harmonic-to-Noise Ratio (H1H2), and creak, were found to be significantly correlated with the HAMD-17 total score. When compared to the HC group, the MDD group exhibited significant reductions in variability in these abnormal speech acoustic features, indicating decreased variability in the MDD group (as shown **Figure 2F**).

### 3.4 Classification model by voice features

#### 3.4.1 Classification model of all associated vocal acoustic features

**Figure 3:**
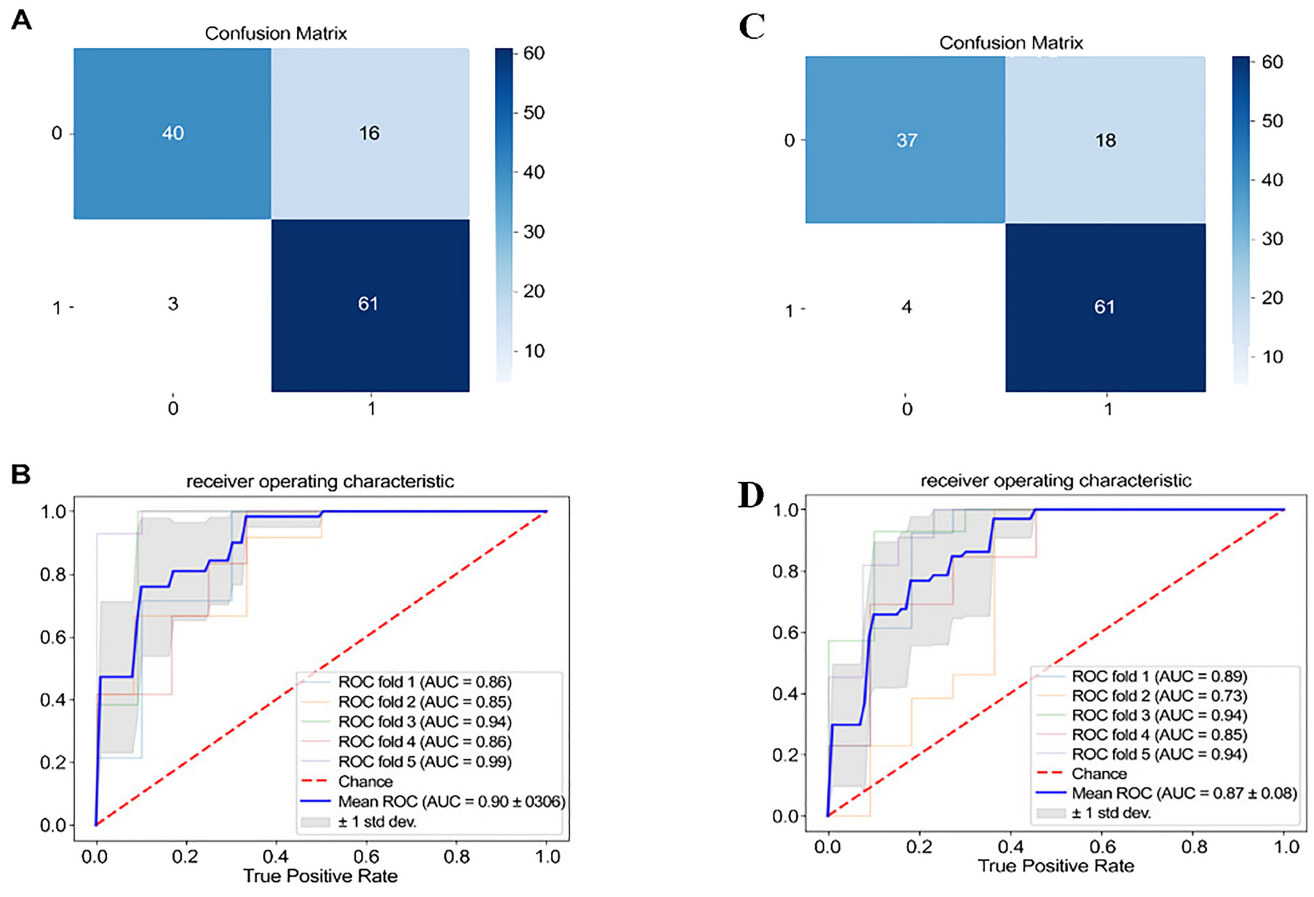
Confusion matrix and ROC (In **A** and **C**, 0 and 1 represent the number of people in the healthy group and the depression group, respectively.).


The confusion matrix of the four-layer neural network classification prediction model revealed that utilizing significant depression-related acoustic speech features distinguished between MDD and HC groups well, with an accuracy rate of 83.33% (as shown **Figure 3A**). The ROC analysis demonstrated that the significant depression-related acoustic speech features for classifying MDD and HC yielded an average AUC of 0.90 ± 0.06 (as shown **Figure 3B**). In this study, the input variables of the classification model consisted of 331 relevant and significant acoustic speech features. In contrast, the output variable represented the two classes: 0 for HC and 1 for MDD. The results of ROC included four outcomes: True Positive (TP), False Positive (FP), False Negative (FN) and True Negative (TN). According to the above matrix results, the performance results of the classification model based on 331 significant vocal acoustic features showed that:

$$\text{Accuracy}\left(\text{ACC}\right)\;=\frac{TP+TN}{TP+FN+FP+TN}=83.33\%$$

$$\text{Error}\left(\text{ERR}\right)\;=\frac{FN+FP}{TP+FN+FP+TN}=\;16.67\%$$

$$\text{Sensitivity}\left(\text{SEN}\right)\;=\frac{TP}{TP+FN}=93.75\%$$

$$\text{Specificity}\left(\text{SPE}\right)\;=\frac{TN}{FP+TN}=71.43\%$$

#### 3.4.2 Classification Model of Top 30 Associated Vocal Acoustic Features

The binary classification results between MDD and HC using the top 30 significantly correlated acoustic speech features are as follows: The confusion matrix demonstrated an accuracy rate of 81.66% when utilizing the top 30 acoustic speech features for classifying MDD and HC (as shown **Figure 3C**). The ROC analysis depicted that using the significant depression-related acoustic speech features for classifying MDD and HC yielded an average AUC of 0.87 ± 0.08 (as shown **Figure 3D**).

### 3.5 Prediction model by voice features

#### 3.5.1 Predictive model with All relevant vocal acoustic features

**Figure 4:**
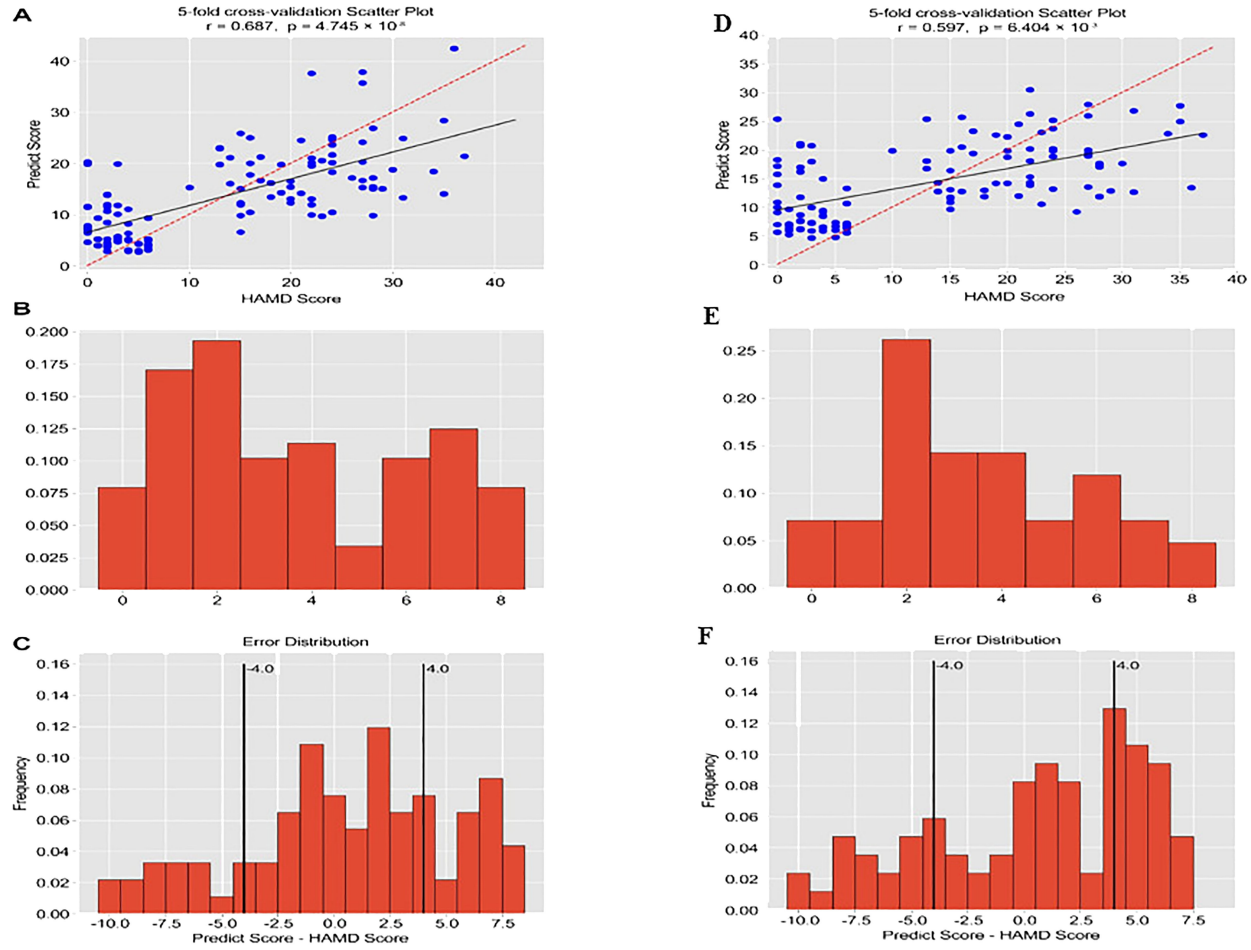
The correlation coefficient between predicted values and actual values, and the prediction error. (The horizontal axis of Figures **B** and **E** represents Mean Absolute Error, and the vertical axis represents frequency).

The four-layer neural network model was utilized to predict the severity scores of depression (HAMD-17) in MDD and HC based on the acoustic speech features. The predicted scores of depression severity were obtained using the 331 relevant and significant acoustic speech features. The scatterplot of the predicted depression severity scores based on acoustic speech features and the actual HAMD-17 scores demonstrated a significant correlation, with a Pearson correlation coefficient of 0.687 and a p-value of 4.745×10^−8^ (as shown **Figure 4A**). The distribution of errors between the predicted scores and actual HAMD-17 scores for all participants was analyzed. The MAE was 4.51 points, and 45.0% of the participants had an MAE below 4.0 (as shown **Figures 4B, C**).

#### 3.5.2 Predictive model with top 30 relevant vocal acoustic features

Further focusing on the prediction model using the top 30 acoustic speech features, the results showed a significant correlation between the predicted depression severity scores based on the top 30 acoustic speech features and the actual HAMD-17 scores. The Pearson correlation coefficient was 0.597, with a p-value of 0.000 (as shown **Figure 4D**). **Figure 4**.3 displays the distribution of errors between the predicted scores and the actual HAMD-17 scores for all participants. The MAE was calculated to be 4.69 points, and 30.83% of the participants had an MAE below 4.0 points (as shown **Figures 4E, F**).

## 4 Discussion

This research utilized computer algorithms to extract the more effective vocal acoustic features associated with the severity of depressive disorders. These features encompassed various rhythm, spectral, and data features (minimum, median, maximum, mean, SD skew, kurtosis, alpha, intercept, and R^2^). Considering the potential complexity of the high-dimensional dataset, the trained model exhibited limited generalizability. Feature dimensionality reduction utilized Pearson correlation analysis with the significance threshold of the p-value less than 0.01. Therefore, 331 acoustic features were found to be significantly associated with the severity of depressive symptoms, indicating vocal acoustic features as the potential biomarkers of depression. These important vocal acoustic features could be utilized to classify and predict MDD and HC effectively. Previous studies primarily relied on linear relationships, which performed t-tests, correlation analysis, and linear regression analysis to investigate the relationship between depressive symptoms and vocal acoustic features Alpert et al. (15). However, limitations exist in the accuracy and stability of extracting and predicting quantitative acoustic feature indicators Ozdas et al. (16)Wang et al. (17). The results demonstrated that the models based on vocal acoustic features could effectively classify and predict the MDD and the HC, and the efficacy of the models was superior to previous research Zhang et al. (6). There were non-linear relationships exist between vocal acoustic features and depressive symptoms.

The main symptoms of depression include low mood, lack of pleasure, and loss of interest. These symptoms can further lead to changes in motor control, resulting in psychomotor retardation. Psychomotor retardation is typically characterized by slowed movements, motor impairments, and poor coordination, which can affect the characteristics of the vocal source and vocal tract Bennabi et al. (18)Goldberg (19). This study also indicated that some dimensions of Peak2RMSs represented the loudness of vocal acoustic features associated with the severity of depressive symptoms. Similar to previous studies, Peak2RMS was significantly smaller in the MDD group compared to the HC Scherer (20); Cohen et al. (21).

Furthermore, the variability in Peak2RMS is significantly higher in MDD than in HC. This result suggests that in addition to mean and median values, the variability (std) of Peak2RMS is also a crucial speech acoustic feature for classifying and predicting MDD and HC. These results were also consistent with the previous research, suggesting that loudness was the crucial vocal acoustic feature of psychomotor retardation in MDD Cannizzaro et al. (22). In contrast, MCEP describes the energy spectrum envelope of the single speech frame and the dynamic information of speech signals. Some evidence suggests that computing MCEP trajectories and incorporating them into the original features could improve speech recognition performance Williamson et al. (23) Consistent with previous research, the dimensions related to MCEP were considered critical biomarkers for diagnosing and identifying MDD.

Additionally, the MFCC_deltas and the MFCC_delta_deltas describing the dynamic changes in speech were significantly associated with the overall depression scores and showed similar trends to MCEP Taguchi et al. (10). This research also supported that MDQ, peakSlope, and Rd conf were associated with the severity of depressive symptoms, which might attributed to the comorbidity of anxiety commonly observed in most MDD Flint et al. (24). MDQ and Rd_conf reflected important vocal acoustic features of emotional changes Cohen et al. (25). The vocal tract of MDD tended to be more tense than HC, and the vocal cord coordination performance was also worse. Analysis of the top 30 vocal acoustic features indicated that MDD exhibited pathological acoustic features with lower volume Flint et al. (24); Cohen et al. (25) Peak2RMSs as loudness ranked second in the top 30 features, indicating a higher weight. MCEP, MFCC_deltas, and MFCC_delta_deltas were all negatively correlated with depressive symptom severity, ranging from -0.563 to -0.611. Consistent with previous research, MCEPs were the essential spectral features for MDD Grabowski et al. (26) Koch et al. (27). Meanwhile, except for regular spectral features, the dynamic trajectory changes in spectral features also showed some significance in predicting the severity of depressive symptoms. The critical vocal acoustic features might be associated with speech retardation, motor impairments, and poor coordination of MDD Trevino et al. (28) Compared to HC, MDD exhibited lower variability in related dimensions of MCEP as the important potential vocal acoustic features for discrimination and predicting MDD and HC Hubbard and Trauner (29). Consistent with previous research findings, the median and mean values of Formants are significantly negatively correlated with depression. This result suggests that individuals with MDD showed lower formant than HC Garcia-Toro et al. (30).

This research conducted a Neural Architecture Search to find the optimized neural network for classifying and predicting depression. Compared to basic low-level features such as means and standard deviations, higher-level vocal acoustic features could improve the model’s predictive ability Grabowski et al. (26). The predictive model also achieved good prediction performance, as indicated by the high correlation between the depression prediction scores of the model and the actual scores obtained from the HAMD-17 assessment (r=0.687, P<0.01). This research calculated the MAE to validate the effectiveness of the predictive model further. The average MAE was 4.51, and 45.83% of the participants had an MAE smaller than 4. Compared to MAE values of 5.36 and 5.07, the predictive model in this research based on neural networks exhibited better predictive effectiveness Fan et al. (31)Al Hanai et al. (32) The 331 critical vocal acoustic features showed better predictive ability for depression using deep learning models, with relatively minor prediction errors.

## 5 Conclusion

This research found 331 important vocal acoustic features associated with the severity of depressive disorders, including MCEP, MFCC_deltas, MFCC_delta_deltas, Formant, Peak2RMS, creak and others. These vocal acoustic features can accurately distinguish participants with MDD from those in the HC group. Furthermore, although the classification model performance showed a small decrease, the top 30 critical vocal acoustic features could discriminate between MDD and HC. Therefore, the classification model based on the vocal acoustic features could be utilized to identify MDD and HC effectively and consistently.

## 6 Limitations

In this study, individuals with MDD exhibit both depressive and anxiety symptoms. The symptoms between the two conditions overlap significantly, and comorbidity rates are high. Comorbidity of depression and anxiety is one of the most common forms of comorbidity in clinical psychiatry. This study’s results may have been influenced by comorbid anxiety. Future research should consider examining the acoustic features of different clinical characteristics and types of disorders in greater depth to optimize the model.

## Data Availability

The raw data supporting the conclusions of this article will be made available by the authors, without undue reservation.
