# Supplementary material for: Enhanced classification and severity prediction of major depressive disorder using acoustic features and machine learning
Source: Front Psychiatry. 2024 Sep 17;15:1422020. doi: 10.3389/fpsyt.2024.1422020 (PMC11442283; doi:10.3389/fpsyt.2024.1422020)
Supplement: Supplementary file 1 [file Table1.pdf]

Supplementary materials

Table 1 Summaries of features

Feature Name		Num
F0	Vocal cord vibration cycle	1
VUV	vector containing the binary voicing decisions	1
NAQ	Normalized amplitude quotient is presented as a method to parametrize the glottal closing phase.	1
QOQ (Rabiner, 2010)	The quasi-open period describes the duration of the glottal flow above 50% of the peak amplitude (Schafer & Rabiner, 1970).	1
H1H2	Difference in glottal harmonic amplitude	1
PSP (Williamson et al., 2016)	Parabolic spectral parameter fitting a parabolic function to the low-frequency part of the estimated glottal flow.	1
MDQ	The Maxima Dispersion Quotient, is proposed for discriminating breathy to tense voice (G. Degottex, 2014).	1
Peak Slope	Slope coefficient of a regression line fit to local peak by using wavelet analysis.	1
Rd (Pedregosa, 2013)	The Rd shape parameter (Alku et al., 2002) of the Liljencrants-Fant (LF) glottal model (T, 1989) using the Mean Squared Phase (MSP) method based on MSPD2 (Morency, 2013) (Alku et al., 1997) (John Kane, 2012).	1
Rd-conf		1
Creak (Kane, 2012)	Detect creaky voice using acoustic features by an artificial neural network.	1
MCEP (Kane, 1995)	Transform the spectrogram into a Mel spectrum through the Mel scale filter bank, and then perform cepstrum analysis.	25
HMPDM (Fant G, 1985)	Harmonic Model Phase Distortion Mean and Harmonic Model Phase Distortion Deviation are flexible representation of the glottal source based on the short-term statistics of the phase distortion.	25
HMPDD (Fant G,		13

1985)		
Peak-to-RMS	Peak-to-RMS measure reflecting a local loudness metric related to waveform shape across a few pitch periods.	1
Formant	Formants refer to areas where energy is relatively concentrated in the sound spectrum.	5
MFCC-deltas	Reflecting the dynamic information of the spectrum envelope on a frame of speech signal.	20
MFCC-delta-deltas		20
